# A Retrospective Study to Assess Temporal Trends in Mortality Related to Liver Disease From 1999 to 2020 in Patients With Depression in the United States

**DOI:** 10.7759/cureus.92579

**Published:** 2025-09-17

**Authors:** Bryan P Vintimilla Herrera, Srikanth Chittareddy, Christopher Williams Valsaint, Leandro Feo

**Affiliations:** 1 Surgery, Independent Research, Little Ferry, USA; 2 Surgery, Malla Reddy Institute of Medical Sciences, Hyderabad, IND; 3 Surgery, Faculte De Medecine et de Pharmacie de I'Universite d'Etat d'Haiti, Port-au-Prince, HTI; 4 Gastrointestinal Surgery, Delray Medical Center, Delray Beach, USA

**Keywords:** age-adjusted mortality rate, cdc mcd, chronic liver disease (cld), depression, liver disease

## Abstract

Introduction: Liver diseases are major causes of mortality, and their association with depression remains underexplored. Understanding this relationship is essential in identifying high-risk populations and developing targeted public health interventions.

Aims: This study aims to analyze mortality trends and demographic disparities in liver disease with depression as a contributing cause, using the Centers for Disease Control and Prevention (CDC) Multiple Cause of Death (MCD) database (1999-2020).

Methodology: A retrospective observational study was conducted using the CDC MCD database to assess mortality trends in individuals aged 25 years and older in the United States from 1999 to 2020. The study included deaths where liver disease (ICD-10: K70-K76) was listed as the underlying cause and depression (ICD-10: F32) as a contributing cause. Data were analyzed by age, gender, race, geographic region, and place of death. Age-adjusted mortality rates (AAMR) and annual percentage change (APC) were calculated.

Results: A total of 3,945 deaths were recorded. The AAMR initially increased (+8.25% APC from 1999 to 2007) and kept increasing slightly (+8.41% APC from 2010 to 2020). The highest mortality was observed in males (N = 2075, 52.6%), White individuals (N = 3647, 92.4%), and metropolitan regions (N = 3142, 79.7%). Temporal trends showed a higher AAMR in females from 2011 to 2020 (+11.99% APC) compared to 1999-2006 (+11.94%).

Conclusions: Overall, AAMR is trending upward. Our findings emphasize the need for targeted prevention strategies and improved healthcare access across all demographics.

## Introduction

Chronic liver disease (CLD) is a major contributor to mortality in the United States, with 52,222 deaths reported in 2023 and a rate of 15.6 per 100,000 population, positioning it as the ninth leading cause of death nationally [1]. Globally, liver disease causes two million deaths each year, half of which result from cirrhosis. In the United States, over 4.5 million adults are living with diagnosed liver disease [2]. The burden is driven by preventable conditions like alcohol-associated liver disease (ALD), non-alcoholic fatty liver disease (NAFLD), and viral hepatitis, with a higher mortality in middle-aged adults, men, Hispanic and American Indian populations, and in the Southern United States [3].

Depression is common among patients with liver disease and is linked to higher overall and cardiovascular mortality, especially in those with NAFLD. It also increases the risk of suicide, regardless of alcohol use or demographics [4]. This overlap contributes to rising deaths from suicide, substance use, and liver disease seen in several US regions [5]. While liver-related mortality has declined in Black populations, it has increased in White populations, highlighting emerging disparities and the need for integrated strategies that address both physical and mental health [4,6].

Liver disease and depression are linked by shared pathophysiological mechanisms, including systemic inflammation, gut-liver-brain axis dysfunction, and metabolic disturbances. CLD promotes gut dysbiosis and increased intestinal permeability, leading to systemic inflammation and neuroinflammatory changes that contribute to depressive symptoms [3,7,8]. Conversely, depression can exacerbate liver disease progression through heightened hypothalamic-pituitary-adrenal (HPA) axis activity and proinflammatory cytokine release, increasing the risk of hepatic decompensation and mortality [8-10]. Analyzing the relationship using the Centers for Disease Control and Prevention (CDC) Multiple Cause of Death (MCD) database is necessary to clarify population-level mortality trends, identify high-risk groups, and inform public health strategies in the context of rising comorbid liver disease and depression.

## Materials and methods

Study design and data source

We conducted a retrospective, population-based observational study using the CDC Wide-Ranging Online Data for Epidemiologic Research (CDC WONDER) MCD database [10]. This publicly available database contains deidentified mortality data derived from US death certificates. Data extraction was performed on July 8, 2025. As the dataset contains no personally identifiable information, the study was classified as nonhuman subjects research and did not require institutional review board approval [11,12].

Study population

Mortality records from January 1, 1999, through December 31, 2020, were included. Eligible cases were individuals aged 25 years and older in whom CLD (ICD-10 codes K70-K76) was recorded as the underlying cause of death, and depression (ICD-10 code F32) was listed as a contributing cause. Records not meeting these criteria were excluded.

Variables

The demographic and clinical variables extracted from the database included sex (male or female) and race/ethnicity, categorized as White, Black or African American, American Indian or Alaska Native, and Asian or Pacific Islander. Geographic characteristics were defined according to the 2013 National Center for Health Statistics (NCHS) Urban-Rural Classification, which distinguishes metropolitan areas (large central metro, large fringe metro, medium metro, and small metro) from nonmetropolitan areas (micropolitan and noncore). The place of death was also recorded and categorized as a medical facility, home, nursing facility, hospice, or other.

Outcome measures

The primary outcome was mortality from CLD with depression as a contributing cause. Mortality rates were age-adjusted to the 2000 US standard population and expressed per 1,000,000 individuals.

Statistical analysis

We used descriptive statistics (absolute numbers and percentages) to summarize the distribution of deaths across demographic and geographic subgroups. To evaluate temporal patterns, we employed Joinpoint Regression Analysis (Joinpoint version 5.3.0.0, November 2024) to calculate annual percentage change (APC) in age-adjusted mortality rates (AAMR) over time. Trends were stratified by sex, race/ethnicity, and geographic category to identify disparities. A two-sided significance level of p < 0.05 was used to define statistically significant APCs.

## Results

Between 1999 and 2020, the CDC’s MCD database reported a total of 3,945 deaths in the United States among individuals aged 25 years and older that met the study criteria. Specifically, these deaths listed liver disease (ICD-10 codes K70-K76) as the primary underlying cause and also noted depression (ICD-10 code F32) as a contributing factor. The crude mortality rate associated with liver disease combined with depression was calculated at 0.9 deaths per 1,000,000 population. Deaths that did not meet these inclusion criteria were excluded from the analysis.

Demographic and geographic characteristics

Out of the total deaths analyzed (Table 1), males represented 2,075 cases (52.6%), while females accounted for 1,870 cases (47.4%). The mortality rate associated with liver disease, with depression noted as a contributing factor, was higher among males than females, suggesting a possible gender disparity in mortality. In terms of racial distribution, White individuals comprised the majority of deaths at 3,647 (92.4%), followed by Black or African American individuals at 180 (4.6%), American Indian or Alaska Native individuals at 78 (2%), and Asian or Pacific Islander individuals at 40 (1%). These findings indicate that the mortality burden was most pronounced among White individuals, underscoring racial differences in death rates associated with liver disease and co-occurring depression. Of all the deaths that occurred, the metropolitan areas had the highest number at 3142 (79.7%), while nonmetropolitan areas had 803 (20.3%) deaths. When coming to the place of death, most deaths occurred in medical facilities at 1451 (36.84%), followed by decedents' homes at 1412 (35.8%), nursing homes/long-term care at 697 (17.7%), hospice facilities at 214 (5.4%), and others at 165 (4.19%).

**Table 1:** Demographic, geographic, and clinical characteristics of liver disease-related deaths with depression as a contributing cause in the United States, 1999-2020

Characteristic	Number of deaths	Percentage
Gender		
Male	2075	52.60
Female	1870	47.40
Race		
American Indian or Alaska Native	78	2.00
Asian or Pacific Islander	40	1.00
Black or African American	180	4.60
White	3647	92.40
Place of death		
Medical facility	1451	36.84
Decedent's home	1412	35.85
Hospice facility	214	5.43
Nursing home/long-term care	697	17.69
Other	165	4.19
Geographic area		
Metropolitan area	3142	79.70
Nonmetropolitan area	803	20.30

Temporal trends

**Figure 1:**
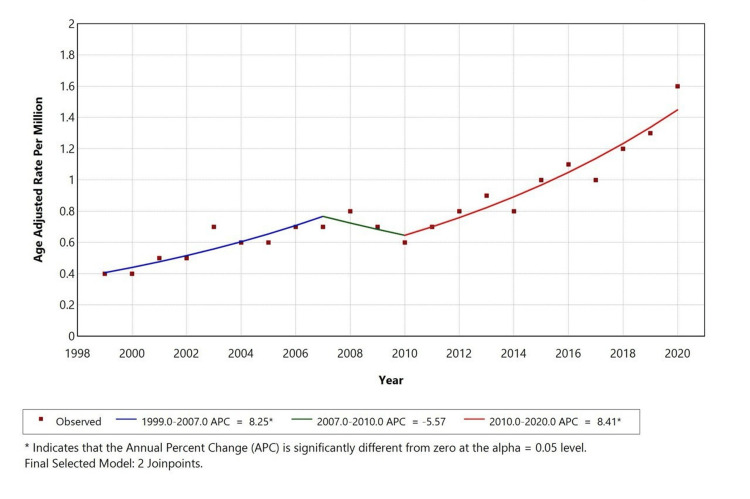
Overall age-adjusted mortality rates among adults aged 25+ in the United States, 1999-2020 *indicates that the annual percentage change (APC) is significantly different from zero at alpha = 0.05 level

During 1999-2020, the AAMR for liver disease with depression as a contributing cause showed an overall increase in trend, with a slight decrease in death rate from 2007 to 2010 when compared to 1999-2007. The APC was 8.25 from 1999 to 2007, -5.57 from 2007 to 2010, and 8.41 from 2010 to 2020 (Figure 1). A similar trend is observed in the White population, with an overall increase in the trend with a slight decrease in death rate from 2007 to 2010 when compared to 1999-2007.

**Figure 2:**
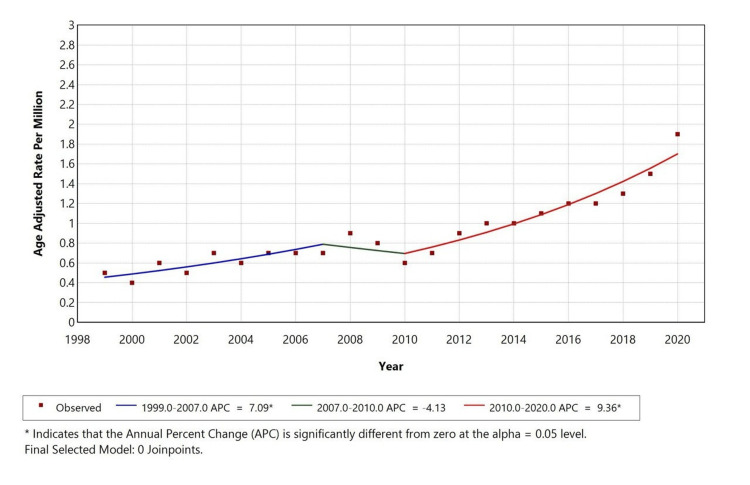
Trends in age-adjusted mortality rates stratified by race among adults aged 25+ years in the United States, 1999 to 2020 *indicates that the annual percentage change (APC) is significantly different from zero at alpha = 0.05 level

The APC is 7.09 from 1999 to 2007, -4.13 from 2007 to 2010, and 9.36 from 2010 to 2020. Significant changes in direction points were observed in the overall population and Whites in 2006 and 2010. Temporal trends for American Indian/Alaska Native, Asian or Pacific Islander, and Black or African Americans are not displayed due to data suppression for counts <10, limiting reliable trend analysis as shown in Figure 2.

**Figure 3:**
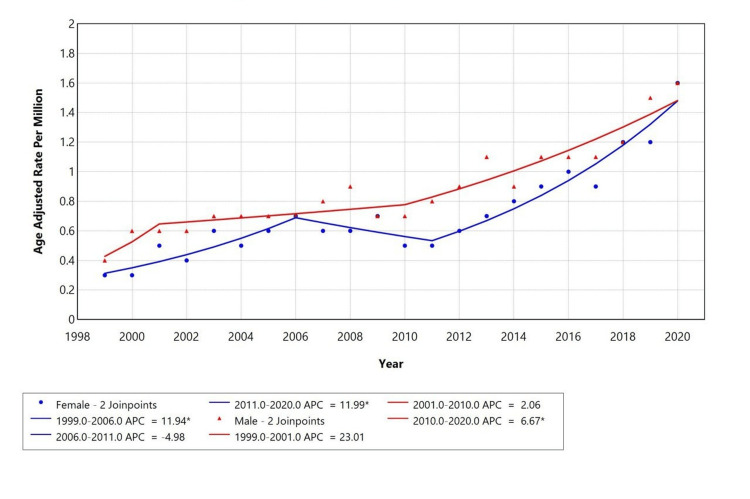
Trends in sex-stratified age-adjusted mortality rates among adults aged 25+ in the United States, 1999-2020

When stratified by gender, there is an increase in trend for both genders from 1999 to 2020, but the female population showed a reverse in trend from 2006 to 2011, while males showed slight stabilization from 2001 to 2010. Males had a higher AAMR compared to females. The APC for females is 11.94 from 1999 to 2007, -4.98 from 2006 to 2011, and 11.99 from 2010 to 2020. The APC for males is 23.01 from 1999 to 2001, -4.98 from 2001 to 2010, and 6.67 from 2010 to 2020 (Figure 3).

## Discussion

A retrospective study was conducted using the CDC MCD database to evaluate mortality trends related to liver disease (ICD-10 codes K70-K76) with depression (ICD-10 code F32) as a contributing cause in the United States from 1999 to 2020, among individuals aged 25 years and older. Our study identified a total of 3,945 deaths where liver disease was listed as the underlying cause, and depression was the contributing factor. Over the selected study period, the AAMR for liver disease with comorbid depression initially increased from 1999 to 2007 (APC: +8.25%), declined from 2007 to 2010 (APC: -5.57%), and then rose again from 2010 to 2020 (APC: +8.41%). We observed that the highest mortality rates were in males (52.6%), White individuals (92.4%), and residents of metropolitan areas (79.7%).

The association between liver disease and depression implicates several mechanisms, such as systemic inflammation, gut-liver-brain axis dysfunction, and metabolic imbalance, and several studies have shown that elevated proinflammatory cytokines like interleukin-6 (IL-6) and tumor necrosis factor (TNF)-alpha may cross the blood-brain barrier and contribute to neuroinflammation, promoting both depressive symptoms and hepatic decompensation [7]. These effects can lead to gut dysbiosis, increased intestinal permeability, and vagal nerve signaling that impair brain function and may have a symbiotic relationship [7,13]. Imaging studies have shown cortical thinning in mood-related regions among patients with steatotic liver disease, enhancing this neurobiological connection [7,13,14]. Depression may also worsen liver outcomes through poor treatment adherence, alcohol use, physical inactivity, and suicide risk [4,15]. Several other factors, such as insulin resistance, neurotransmitter imbalance, and low brain-derived neurotrophic factor (BDNF), are also implicated in the pathogenesis of depression [8,16,17].

Our findings align with existing evidence of an increased mortality burden among patients with comorbid liver disease and depression in the United States. These associations underscore the potential value of integrating mental health care into hepatology services and of developing targeted public health interventions and policies to address this dual burden.

The crude mortality rate in our study was 0.9 per 1,000,000 population; this low rate is similar to prior studies that show an increased liver-related mortality in the presence of depression. As evidenced by a large Korean cohort, a twofold increase in liver-related death among individuals with depressive symptoms (adjusted HR: 2.00; 95% CI: 1.10-3.63) was reported, increasing fourfold in HBsAg-positive patients [18]. Similarly, a UK-based meta-analysis found significantly elevated liver disease mortality risk in individuals with high psychological distress (adjusted HR: 2.59; 95% CI: 1.82-3.68) [15]. These consistent findings across populations reinforce the global relevance of our results when evaluating this disease in other countries.

Although our data showed more deaths among males, current literature suggests that females have a higher prevalence of depression and psychiatric comorbidities; meanwhile, males show greater all-cause and unnatural-cause mortality in this context [19,20]. We also observed high mortality among White individuals (92.4%); this big difference is consistent with studies that have shown higher rates of depression in this population. However, depressed White patients also have a higher mortality risk when compared to Black Caribbean, Black African, and South Asian patients, who show lower hazard ratios for mortality in several studies [19-21]. Socioeconomic status further modifies this relationship, particularly among White individuals in low-income groups [21,22]. These disparities point to the need for culturally sensitive, demographically tailored interventions.

Our results revealed that nearly 80% of deaths occurred in metropolitan areas and that most patients died in medical facilities (36.8%) or at home (35.8%), and only 5.4% in hospice settings. These findings have a similar pattern when compared to Altaii et al., who reported that 49.1% of patients with cirrhosis died in hospitals, 24.1% at home, and 6.4% in hospice [23]. Our study shows a higher rate of home deaths and lower hospice utilization, despite including patients with depression, a known barrier to hospice referral. The differences in place of death between our study and that of Altaii et al. may be attributed to variations in study design and different populations, as Altaii et al. focused on cirrhosis-related deaths, while our study included liver disease with depression as a comorbidity. Thus, the presence of depression in patients with liver disease may influence end-of-life preference, and therefore, increase home deaths and reduce hospice use. The majority of deaths in our study were in metropolitan areas, particularly among Black and White people; our findings align with Wang et al., who reported similar geographic disparities in liver disease mortality [6]. These patterns highlight the need for improved access to palliative and mental health services across urban care settings.

We observed a consistent rise in AAMR for liver disease with depression, with inflection points in 2006 and 2010 that reflect national trends of an increasing mortality in nonalcoholic fatty liver disease (NAFLD) and ALD, with APCs that range from 0.29% to 18.3% depending on etiology [24,25]. There was an increase in cirrhosis-related deaths during the COVID-19 pandemic, going up to 11.25% from 2019 to 2021 each year [26]. These findings suggest our population-level trends reflect broader epidemiological patterns and underscore the compounded impact of mental health on liver-related outcomes.

Clinicians should proactively screen for depression during the management of liver disease, especially in male and White patients, and consider early mental health or palliative referrals in selected patients. Additionally, the public health strategies should address treatment disparities and regional gaps in access to integrated liver disease management and mental health services while promoting policies focused on interdisciplinary care, mental health parity, and equitable access in urban areas, which are crucial to reduce the rising burden of comorbid liver disease and depression.

## Conclusions

This study reveals an overall increasing trend in AAMRs for liver disease with depression as a contributing cause in the US from 1999 to 2020. Given our findings of higher mortality among males, White individuals, and residents of metropolitan areas, public health strategies should prioritize early depression screening and equitable access to mental health services in these high-risk groups. Addressing treatment inequities identified across demographic and geographic subgroups is essential. Future research should focus on clarifying the mechanisms underlying these disparities, evaluating culturally tailored interventions, and examining how regional and systemic factors shape outcomes in patients with comorbid liver disease and depression. Future research should focus on understanding the mechanisms behind these disparities, evaluating culturally tailored interventions, and exploring how regional and systemic factors influence outcomes in patients with comorbid liver disease and depression.

## References

[1] (2025). National Center for Health Statistics: deaths and mortality. https://www.cdc.gov/nchs/fastats/deaths.htm.

[2] Asrani SK, Devarbhavi H, Eaton J, Kamath PS (2019). Burden of liver diseases in the world. J Hepatol.

[3] Sepanlou SG, Safiri S, Bisignano C (2020). The global, regional, and national burden of cirrhosis by cause in 195 countries and territories, 1990-2017: a systematic analysis for the Global Burden of Disease Study 2017. Lancet Gastroenterol Hepatol.

[4] Le Strat Y, Le Foll B, Dubertret C (2015). Major depression and suicide attempts in patients with liver disease in the United States. Liver Int.

[5] Tapper EB, Parikh ND (2018). Mortality due to cirrhosis and liver cancer in the United States, 1999-2016: observational study. BMJ.

[6] Wang Y, Huang Y, Antwi SO, Taner CB, Yang L (2024). Racial disparities in liver disease mortality trends among black and white populations in the United States, 1999-2020: an analysis of CDC WONDER database. Am J Gastroenterol.

[7] Kronsten VT, Tranah TH, Pariante C, Shawcross DL (2022). Gut-derived systemic inflammation as a driver of depression in chronic liver disease. J Hepatol.

[8] Ntona S, Papaefthymiou A, Kountouras J (2023). Impact of nonalcoholic fatty liver disease-related metabolic state on depression. Neurochem Int.

[9] Kim D, Manikat R, Shaikh A, Cholankeril G, Ahmed A (2024). Depression in nonalcoholic fatty liver disease and all-cause/cause-specific mortality. Eur J Clin Invest.

[10] Huang X, Liu X, Yu Y (2017). Depression and chronic liver diseases: are there shared underlying mechanisms?. Front Mol Neurosci.

[11] (2025). National Center for Health Statistics: mortality data on CDC WONDER. https://wonder.cdc.gov/mcd.html.

[12] (2025). Office for Human Research Protections: coded private information or specimens use in research, guidance (2008). Published.

[13] Smith ML, Wade JB, Wolstenholme J, Bajaj JS (2024). Gut microbiome-brain-cirrhosis axis. Hepatology.

[14] Arold D, Bornstein SR, Perakakis N, Ehrlich S, Bernardoni F (2024). Regional gray matter changes in steatotic liver disease provide a neurobiological link to depression: a cross-sectional UK Biobank cohort study. Metabolism.

[15] Russ TC, Kivimäki M, Morling JR, Starr JM, Stamatakis E, Batty GD (2015). Association between psychological distress and liver disease mortality: a meta-analysis of individual study participants. Gastroenterology.

[16] Lee JW, Park SH (2021). Association between depression and nonalcoholic fatty liver disease: contributions of insulin resistance and inflammation. J Affect Disord.

[17] Radford-Smith DE, Patel PJ, Irvine KM (2022). Depressive symptoms in non-alcoholic fatty liver disease are identified by perturbed lipid and lipoprotein metabolism. PLoS One.

[18] Cho IY, Chang Y, Sung E (2020). Depressive symptoms and risk of liver-related mortality in individuals with hepatitis B virus infection: a cohort study. Scientific Reports.

[19] Patel P, Ali H, Inayat F (2023). Racial and gender-based disparities and trends in common psychiatric conditions in liver cirrhosis hospitalizations: a ten-year United States study. World J Hepatol.

[20] Das-Munshi J, Chang CK, Schofield P, Stewart R, Prince MJ (2019). Depression and cause-specific mortality in an ethnically diverse cohort from the UK: 8-year prospective study. Psychol Med.

[21] Cui Y, Zheng W, Steinwandel M, Cai H, Sanderson M, Blot W, Shu XO (2021). Associations of depressive symptoms with all-cause and cause-specific mortality by race in a population of low socioeconomic status: a report from the southern community cohort study. Am J Epidemiol.

[22] Kardashian A, Serper M, Terrault N, Nephew LD (2023). Health disparities in chronic liver disease. Hepatology.

[23] Altaii H, Al-Kindi SG, Yaqoob Z, Al-Khazaraji A, Romero-Marrero C (2018). Place of death and hospice utilization among patients who die from cirrhosis in the United States. Clin Gastroenterol Hepatol.

[24] Paik JM, Golabi P, Younossi Y, Mishra A, Younossi ZM (2020). Changes in the global burden of chronic liver diseases from 2012 to 2017: the growing impact of NAFLD. Hepatology.

[25] Paik JM, Shah D, Eberly K, Golabi P, Henry L, Younossi ZM (2024). Changes in mortality due to chronic liver diseases (CLD) during the COVID-19 pandemic: data from the United States' National Vital Statistics System. PLoS One.

[26] Fedeli U, Barbiellini Amidei C, Casotto V, Grande E, Saia M, Zanetto A, Russo FP (2023). Mortality from chronic liver disease: recent trends and impact of the COVID-19 pandemic. World J Gastroenterol.

